# Influence of Carbon Nanotubes and Its Derivatives on Tumor Cells In Vitro and Biochemical Parameters, Cellular Blood Composition In Vivo

**DOI:** 10.1186/s11671-018-2689-9

**Published:** 2018-09-12

**Authors:** Olena M. Perepelytsina, Andriy P. Ugnivenko, Alexey V. Dobrydnev, Olga N. Bakalinska, Andrii I. Marynin, Mychailo V. Sydorenko

**Affiliations:** 1 Department for Biotechnical Problems of Diagnostic IPCC, Nauky str.,42/1, Kiev, 03028 Ukraine; 2 Chemistry Department, Taras Shevchenko National University of Kiev, Lva Tolstoho str. 12, Kiev, 01033 Ukraine; 3 Chuiko Institute of Surface Chemistry NAS of Ukraine, 17 General Naumov str., Kiev, 03164 Ukraine; 4 National University of Food Technologies, 68, Volodymyrska str., Kiev, 01601 Ukraine

**Keywords:** Multi-walled carbon nanotubes, Nanotoxicity, Multicellular tumor spheroids, Doxorubicin, Hepatic function, Cell blood composition, 61.46+w61.48+c61.48De87.15-v87.64-t

## Abstract

The aim of the proposed work was to analyze the toxicity of oxidized carbon nanotubes (CNTox), functionalized by doxorubicin (CNT-Dox) and fluorescein (CNT-FITC) on cell and organism level. The cytotoxic effect of CNTox, CNT-Dox, and CNT-FITC was analyzed on tumor cells in vitro (2-D, 3-D cultures) and on Balb2/c mice model in vivo. As a result, it was demonstrated the possibility of doxorubicin immobilization on the surface of CNT and controlled release of doxorubicin (Dox) from the surface of CNT. Dox immobilization coincident with decreasing cytotoxic effect CNT-Dox compared with free Dox. Breakdown of peptide bonds with CNT surface led to the release of doxorubicin and dose-dependent enhancement of the cytotoxic effect of CNTs and Dox. The combined cytotoxic effect from CNTs, Dox, and trypsin on the survival of tumor cells was shown. At the organism level, it was investigated the effect of the obtained nanostructures on the state of hepatic enzymatic system, the protein metabolism, and cell blood composition of the experimental animals. CNTox influence in vivo model was statistically the same as control. CNT-Dox demonstrated lower total organism toxic effect compared to the pure doxorubicin. Deviations in the cell blood composition indicated a general toxic effect of CNT-Dox, but it was more moderate compared with of pure doxorubicin. From the data obtained, we concluded that binding CNTs with doxorubicin allows reducing toxicity of the doxorubicin on the general biochemical indicators of blood and violations in the blood cells composition in vivo. At the same time, the combined effect of CNTs and doxorubicin after drug release allowed us to achieve greater efficacy in suppressing tumor growth in vitro.

## Background

One of the most striking and promising applications of carbon nanotubes is medicine. Carbon nanotubes (CNTs) are characterized by unique chemical and biological properties [1–5]. CNTs have a large surface area that allows them to attach a wide range of biological substances [6]. In addition, CNTs are able to penetrate through cell membranes, capillaries, and accumulated in cells and tissues [7–9]. CNTs are absorbed by cells easily and it promotes CNTs’ ability to reach cell nuclei and implies the possibility of gene therapy [10]. That is why CNTs are attractive vehicles for the transport of proteins, antigens, RNA/DNA vectors [11], vaccines, and drugs into the cells [12]. Particular interest is in the prospects for use of CNTs as personalized carrier with controlled drugs release for anticancer [13], antibacterial [14], and immunological therapy [15]. Targeted delivery and controlled release is the actual problem of the modern use of drugs, especially with cytotoxic properties. The exact mechanism of drug release ensures an effective concentration of the active substance in the target tissue with a minimal concentration in others. It would reduce the dose of the drug with maintaining its effectiveness and diminution of harm from side effects. Currently, there are various ways of purposeful drug delivery, such as delivery using liposomes [16], polymeric micelles and dendrimers [17], biodegradable particles [18], and other nanoparticles [19]. But recent experiments have demonstrated a lot of benefits from using CNTs in delivering drugs compared to other nanoparticles [20, 21]. One of them is that CNTs with fairly large and active surface that can be filled with the desired chemical substance, ranging from small molecules to proteins, antibodies, and RNA/DNA. Open ends of CNTs make the inner volume and surface accessible for functionalization. So, the large surface area of CNTs provides a lot of binding sites for different type of functionalization. At the same time with prospects of CNTs, there are some problems with low solubility of CNTs, ability for aggregation, hydrophilic qualities, long half-life, and influence on whole organism [22]. According to the literature, the introduction of CNTs into the body of experimental animals is accompanied with accumulation of CNT and its derivatives in the organs of the digestive tract, spleen, kidneys [23], muscles [24], and lung tissue [25]. On the next step, CNTs influence on the activity of metabolic and inflammatory processes [25, 26], state of immunity system [27], and survival of experimental animals [28]. Nevertheless, these problems are the subject of active research for further advancement in the use of carbon nanotubes. The advantages of CNTs as nano-vectors for drug delivery have been convincingly demonstrated in a number of studies. Authors of [29, 30] reported that specific nanotubes may be less harmful than nano-carriers for drugs. It has also been shown that simple functionalization (-OH, -COOH, -NH, PEG) [31] or encapsulation of the CNTs [22], increases the water solubility, improves bioavailability, and reduces toxicity of CNTs. And the introduction of CNTs to mice with a transplanted tumor may be effective against targeted transformed tissue and led to decreasing of tumor volume and to increasing animal survival [32].

At the base of previous investigations [33], we assumed that active antitumor substance (doxorubicin) could be immobilized on the surface of carbon nanotube peptide bonds. The resulting compound, carbon nanotubes which were functionalized by doxorubicin (CNT-Dox), could reduce cytotoxic properties of CNT as doxorubicin (Dox). At the same time, the disintegration of the treated construct may allow to realize the cytotoxic properties CNT and Dox. Thereby, it is possible to achieve increasing the antitumor activity of both substances. Therefore, the aim of present study was to determine the cytotoxic effect of CNTs-Dox construct in the presence of protease (trypsin) in vitro. We analyzed the impact of CNTs and its derivatives on tumor cells in 2D and 3D cell model, in vitro. The other aim was to investigate the influence of the obtained compounds (CNTs, CNTox, and CNT-Dox) on the activity of hepatic enzyme system, proteins turnover, and on cellular blood composition in vivo. So, the authors realized a complex estimation of the toxicity, bioavailability, and effectiveness of the use of CNTs and their derivatives against tumors of gastrointestinal tract.

## Methods

Cell line of Caucasian colon adenocarcinoma grade II carcinoma (HT29) was used as experimental cell model in 2D (monolayer) and 3D (spheroid) system in vitro. The line was purchased from Bank of cell lines and tissues of animals Kavetsky’ Institute of experimental pathology, oncology, and radiobiology NAS Ukraine. The cells were handled in standard cell culture conditions (95% humidity, 5% CO_2_ in air; 37 °C) in DMEM full medium with 10% FBS under laboratory containment level 2.

### Synthesis, Oxidation, and Functionalization of CNT

The initial Multi-walled carbon nanotubes (MWCNTs) were obtained by chemical vapor deposition (propylene and hydrogen with Mo/Fe/Al_2_O_3_ as catalyst). Synthesized MWCNTs had 10–30 layers, internal diameter 5–15 nm, external diameter 10–60 nm, length 20–500 μm, specific surface area 120 m^2^/g and bulk density 5–50 g/l [33].

Purification of MWCNTs from the metal/catalyst was performed by HF treatment. Elimination of amorphous carbon from carbon nanomaterials was realized by oxidation in air at 450–500 °C. The crude sample containing MWCNTs reacted with oxygen from air and created carbon dioxide or carbon monoxide. Some amount of carbon deposit has been obtained after HF (aqua) dissolution of the catalyst support was oxidized in air at 450–500 °C. An air flow quartz tubular reactor was used for this procedure for 130–150 min. We analyzed and described physical and chemical characteristics of obtained MWCNTs in previous work [33].

Scanning electron spectroscopy (SEM) of MWCNTs (JEOL SL6060LA, Japan) was used to determine the structural characteristics of MWCNTs. After immobilization of the ligands, the morphology was not changed.

### Oxidation of MWCNTs

On the next step, the purified carbon nanotubes (CNTs) were oxidized in 70% HNO_3_ at 99 °C for 4 h and then washed with distilled water and 10% NH_4_OH solution for 12 h. As a result, carbon nanomaterial oxides MWCNTox (CNTox) were synthesized. After that, CNTs were washed thrice with dH_2_O to neutral pH. The acidic sites on CNT surface were regenerated with 0.1 M HCl solution. The resulting oxidized CNTs-ox were separated by centrifugation, rinsed extensively, and re-suspended in dH_2_O water.

### Preparation of CNT-Dox Particles

**Fig. 1:**
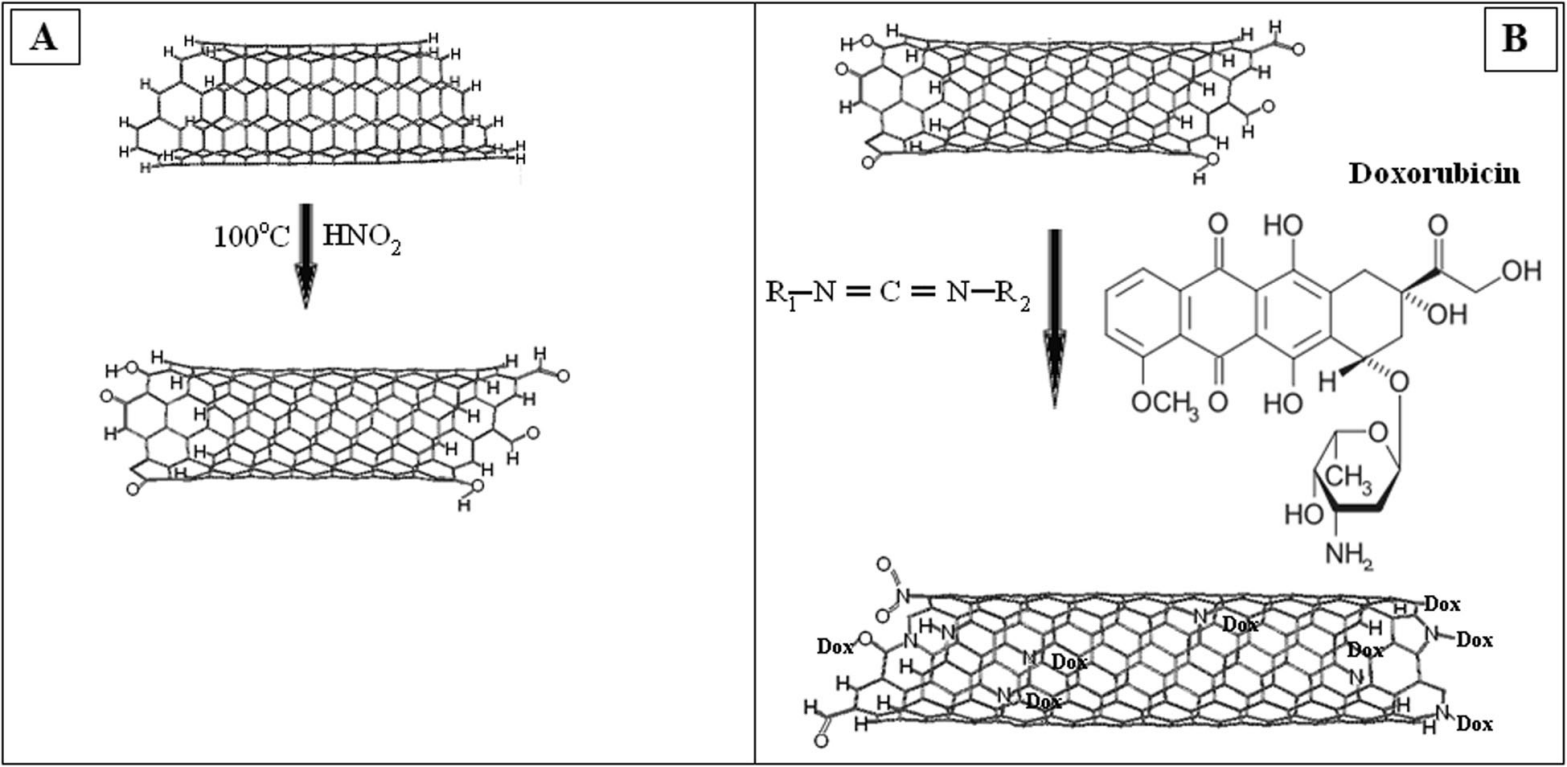
**a** Scheme of reaction of CNTs with carbodiimide. **b** Scheme of the functionalization of the CNTs by doxorubicin

**Fig. 2:**
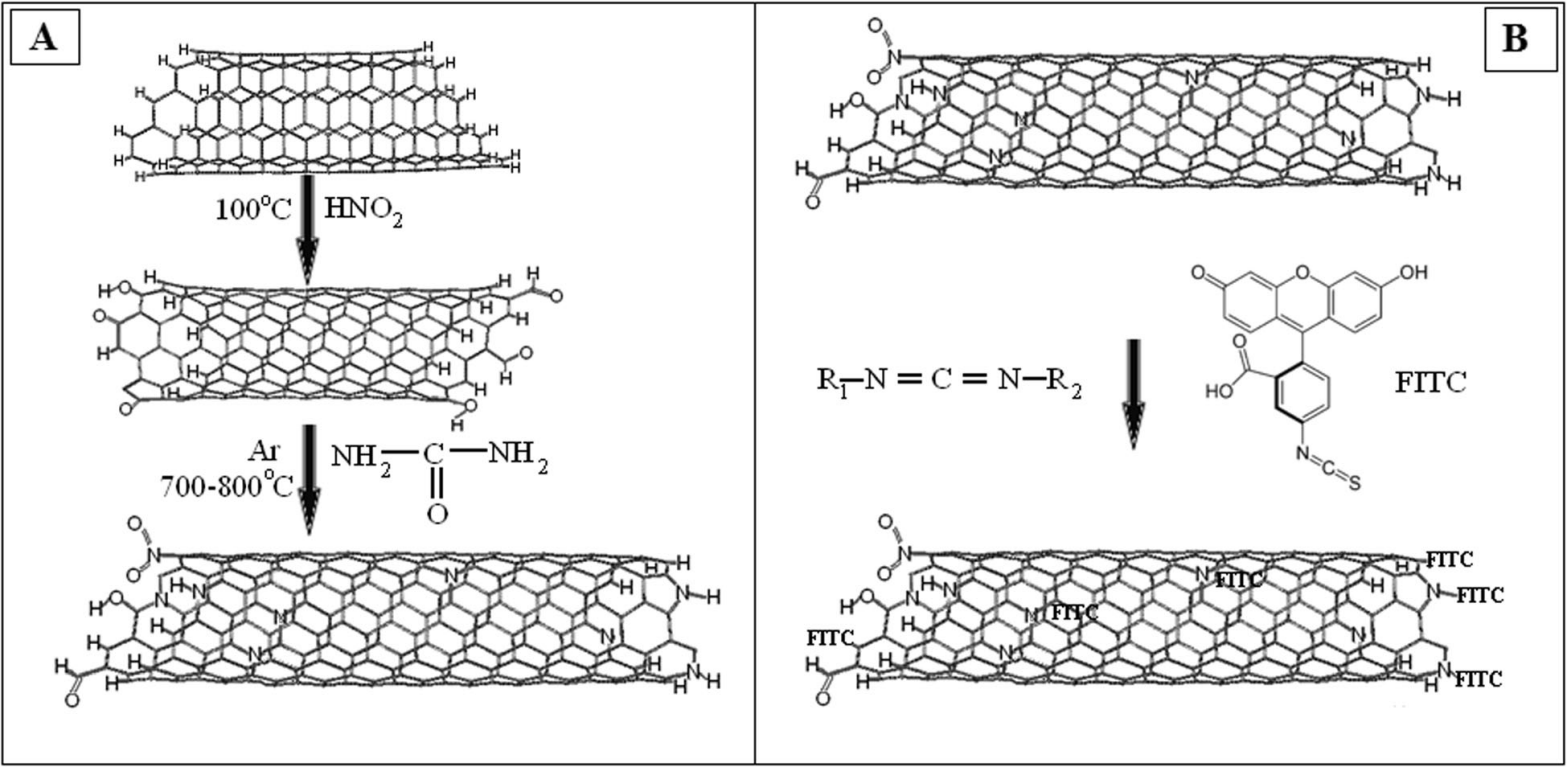
**a** Scheme of reaction of CNTs with urea. **b** Scheme of the functionalization of the CNTs by FITC

The task of the next step was to immobilize Dox hydrochloride (Dox, Teva Nederland B.V, Netherlands) on the surface of MWCNTs particles. To functionalize the surfaces of oxidized CNTs (200 mg) by amino containing Dox-lactose monohydrate, they were incubated with bifunctional linking agent—N-cyclohexyl-N′-(2 morpholinoethyl) carbodiimide metho-p-toluenesulfonate (C 1011, Sigma Aldrich, USA) during 15 min at room temperature (Fig. 1a). After that, 100 mg DOX-lactose monohydrate was added into 10 ml of 0.15 M phosphate buffer pH 6.5 to each nanocarbon materials. The mixture was allowed to react at 30 °C for 24 h. In such conditions, covalent binds between Dox and CNTs were formed (Fig. 1b). The composition were collected by centrifugation at 10,000 rpm 15 min and washed with dH_2_O thrice. Supernatants were used for detection concentration of free DOX by spectrophotometer. Immobilization of FITS on CNT surface was provided by scheme shown on Fig. 2a, b.

### Preparation of Stable Suspensions of MWCNTs

The colloidal suspension of MWCNTs was carried out in two stages. In the first stage, carbon nanomaterials have been subjected to ultrasonic treatment in phosphate saline buffer (PBS) using an ultrasonic disperser UZDN—2 T. The processing modes were *I* = 10 mA, *R* = 22 kHz, duration—30 min. In the second stage, the resulting hydrosol was dispersed by the centrifugation at room temperature. The process includes several centrifugation cycles. So, the hydrophilic dissolving SWCNTs fraction was selected this way. Before adding to the suspension cultured cells, solutions of MWCNTs were sterilized by boiling during 30 min. Derivatives of CNTs (CNT-Dox and CNT-FITC) were sterilized by 100-fold concentration of PSGA (Penicillin: Streptomycin: Gentamycin: Amphotericin).

### Zeta Potential of Suspensions of Nanotubes

Zeta potential of samples were measured by electrophoretic light scattering (M3-PALS). For measurements, the Zetasizer Nano ZS analyzer, manufactured by Malvern Instruments (Malvern, UK), was used. Experiments were carried out at 25 °C with seven repeats. To measure the samples, a universal submersible electrode (Universal Dip Cell) ZEN1002 and polystyrene cuvettes (DTS001), a light source—an H-Ne 633 nm laser, were used.

### FTIR Spectroscopy

The chemical composition of the obtained nanomaterials was investigated by Fourier-transform infrared (FTIR) spectroscopy. IR spectra were determined by FTS 7000e Varian FTIR spectrometer. Samples for analysis were prepared by grinding in a mill a mixture of ~ 1 mg of CNTs and 150 mg of spectrally pure KBr. Samples were prepared by using a press with a pressure force of 3.0–3.5 × 10^3^ kg/cm^2^. The samples were dehydrated by heating at a temperature of 600 °C for 60 min. Pre-shot spectra of KBr were preliminary obtained, after that they were subtracted from the spectra of the samples. All spectra was analyzed according catalog of Spectrometric Identification of Organic Compounds [34]. For comparison, samples of MWCNTs oxidized (CNT-O), MWCNTs functionalized by doxorubicin (CNT-D), and MWCNTs functionalized by fluorescent label (CNT-F) were used. All processing and conditions, of samples and concentration, were the same for three materials.

### Colorimetric Assay of Doxorubicin Release from CNTs Surface

To evaluate the amount of free Dox after immobilization on CNMs and the effectiveness of the Dox release, the ability of free Dox to fluorescent at a wavelength of 495 nm was used [35]. Active concentration of Dox in Dox hydrochloride was 16.7% *w*/*w*. For the purpose of drawing the calibration lines, Dox Teva 20 mg/ml with ten dilutions to 8 × 10^−3^ mg/ml was used. Then, the fluorescence of free Dox was measured in supernatant from complexes DOX with CNTs by spectrophotometric plate reader Multiscan (Labsystem, Finland).

### Functional Groups Concentration in CNT-DOX, CNT-FITC Particles

For Dox immobilizations, 1 g of MWCNTs in 1 ml dH_2_O was used. The amount of Dox-TEVA was 100 mg of (16.7 mg of active Dox) in 1 ml dH_2_O. The amount of free Dox in supernatant after reaction was 0.37 mg or 2.2% from active substances. Thus, we made the conclusion that 16.23 mg of DOX was immobilized on 1 g of CNTox. For FITC percent of immobilization was low. Then, 8.35 mg of FITC was immobilized on 1 g of CNTox in 1 ml dH_2_O. Effectiveness of functionalization was 1.62% *w*/*w* for CNT-DOX and 0.84% *w*/*w* for CNT-FITC. Further calculation of concentrations CNT-Dox and CNT-FITC are based on these data.

### Cytotoxic Assay of CNT Derivatives on 2-D and 3-D Cell Model

The cytotoxicity of Dox, CNTs, CNT-FITC, and CNT-Dox was evaluated against HT29 tumor cell using MTT assay. MTT test based on conversion of 3-[4,5-dimetltiazol-2]-2,5-dipheniltetratetrazolium salts to formazan crystals by NAD(P)H-dependent mitochondrial oxidoreductase enzymes in alive cells. Protocol was described by T. Mosmann [36]. In brief, 1 × 10^4^ HT29 were seeded in 96-well plates and cultured in full culture medium for 12 h. Then, current culture medium was replaced by culture medium containing CNTox (sample #1), MWCNTs (sample #2), CNT-Dox (sample #3), Dox (sample #4), and CNT-FITC (sample #5). Concentrations of samples # 1, 2, 3, 5 were 12.5–25–50–100–200 μg/ml, stock concentration of sample #4 was 20 μg/ml, end concentration from 1 to 10 μg/ml. Cells were cultured in full medium and were used as control. After 24 h of incubation, cells were analyzed with MTT by colorimetric assay. To 100 μl of cells suspension, we added 20 μl MTT solution (5 mg/ml PBS, Sigma). After that, cells were incubated with MTT during 4 h in standard conditions. Then, samples were centrifuged under 1500 g, during 5 min, and supernatant was extracted. In all, wells were added 10 μl DMSO (Sigma) for MTT crystals dilution and 20 μl of 25 mM glycine. The absorbance of reacted solution was measured at 540 nm on spectrophotometric plate reader Multiscan (Labsystem, Finland).

### Multicellular Tumor Spheroids Generation

HT29 cells multi-cellular tumor spheroid (MTS) (3D culture) as model system of tumor micrometastasis was cultured by well-established method which was described earlier [33]. Briefly, cell suspension were counted using Trypan blue and planted an equal number of cells (5 × 10^4^ cells/ml). The 3D cell culture was maintained in DMEM (Sigma, USA) medium with 10% FBS (Sigma, USA) in standard conditions (95% humidity, 5% CO_2_ in air, 37 °C). Generation of MTS was performed by technology which was developed in our laboratory. The cultivation of tumor cells were maintained for 24 h in 24-well plates coated with 1% agar in culture medium with 0.24% of carboxy-methyl-cellulose. For investigating the dependence of the size and number of MTS on the concentration and type of CNTs, MTS was generated in the presence of various concentrations of CNTs. Before MTS generation to the cell cultures, CNTs solution in PBS was added in culture to the end concentration as described previously for MTT assay. Further cultivation was conducted during 48 h at a constant rotation of plates on orbital shaker (80 rpm). At the next stage, micro photo images were taken by “dark field” method. Overall, more than 120 images were done. Then, the volume of all MTS, which were on the files, was calculated with the use Axio Vision Rel 4.7 program, Zeiss. We used the formula of Rolf Bjerkvig: *V* = 0.4 ∙ *a* ∙ *b*^2^, where geometric sizes of the spheroids (*b* < *a*) [37]. The visualization of the results was carried out on a Stemy 2000C microscope, Zeiss.

### Analysis of the Impact of CNTs on Hepatic Enzyme System, Proteins Turnover, and on Cellular Blood Composition In Vivo

The procedures involving the animals and their care were conformed to the European directive 2010/63 EU, approved by the local Animal Experimentation Ethics Committees (Protocol №1 21.10.2016). In order to analyze the influence of the obtained substances on the general condition of the organism homeostasis, a series of experiments in vivo was conducted. In vivo studies mice of the Balb/2a line were used. Male and female mice were equal in the groups, aged 6–8 weeks, ten for each group. Mice were housed in cages with steel wire tops and corn-cob beddings and maintained in a controlled atmosphere with 12/12 dark/light cycle, temperature of 22 °C ± 3 °C, and humidity of 50–70%, with free access to food and fresh water. Thus, four groups of mice were formed. Group 1: intact animals were treated with 200 μl of PBS, control. Group 2: mice were treated with CNTox in dose 1.5 mg/kg. Group 3: mice were treated with CNT-DOX in dose 1.5 mg/kg. And in group 4, mice were treated doxorubicin in dose 20 mg/kg. Mice received CNT parenteral in 200 μl of PBS, every 3 days, during 4 weeks. Doxorubicin was administered intraperitoneally, once every 3 days, at a concentration of 20 mg/kg body weight.

### Serum Biochemical Analysis

One month later, mice were withdrawn from the experiment. Cardiac blood samples were immediately collected from dead animals. Blood was taken from animals at the same time from 10 to 11 a.m. For plasma, blood was incubated for 40 min at 37 °C and then centrifuged (20 min, 2000 rpm). Then biochemical parameters, total protein, albumin, aspartate aminotransferase (AST) and alanine aminotransferase (ALT), alkaline phosphatase (ALP), were determined in the plasma with diagnostic kits (Cormay, Warsaw, Poland). Experiments were carried out by unified laboratory protocols on the semi-automatic biochemical analyzer FP-901M (Labsystems, Finland). Cellular blood composition was determined on a hematological analyzer Mindray BC-3000 Plus, China.

### Statistical Analysis

For statistical analysis of 3D culture, all cell aggregates were sorted into groups according to size from 1 × 10^−4^ mm^3^ to 1 × 10^−2^ mm^3^ with step in 1 × 10^−3^ mm^3^. Then number on MTS in each group and median of MTS volume for each group were estimated. All measurements were repeated three times. For micro statistic assay normally distributed random variables, we used the Student’s coefficient for small population. The indicated *p* value was *р ≤ 0.05 or **р ≤ 0.01.

## Results and Discussion

### Scanning Electron Microscopy of MWCNTs

**Fig. 3:**
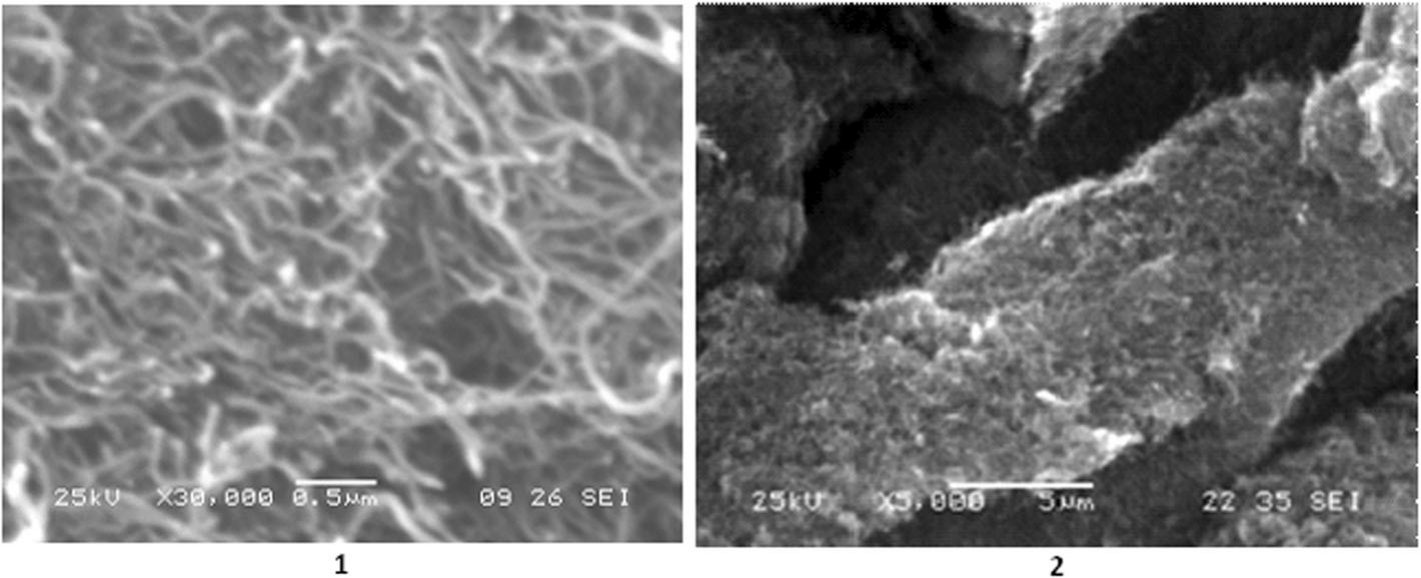
SEM images of CNTs, 1) scale 0.5 μm; 2) scale 5 μm

According to protocol [38], the average diameter of CNTs was 10–20 nm and specific surface area determined by argon desorption was 200–400 m^2^/g. Bulk density within 20–40 g/dm^3^. In particular, the agglomerates in the form of the entangled tubes with dimensions of 20–500 μm are obtained during an industrial CNT production applying CVD method (Fig. 3).

### Zeta Potential of Suspensions of Nanotubes

CNTox and CNT indicate significant stability to aggregation, which directly depends on the value of the zeta potential. CNT-DOX has a smaller zeta potential than CNTox and CNT, and high reproducibility of the measurement, which indicates the homogeneity of the particles (Table 1). A small value of the zeta potential of CNT-FITC can indicate significant particle sizes or their small concentrations, as indicated by the high noise ratio at measurement.

**Table 1:** Zeta potential of CNT samples at dilution 1:10

Sample	Zeta potential, mV
CNTox	− 27.0 ± 2.42
CNT	− 28.7 ± 1.51
CNT-DOX	− 13.9 ± 0.67
CNT-FITC	− 2.24 ± 1.17

### FTIR Spectroscopy of CNTs, CNT-Dox, and CNT-FITC

**Fig. 4:**
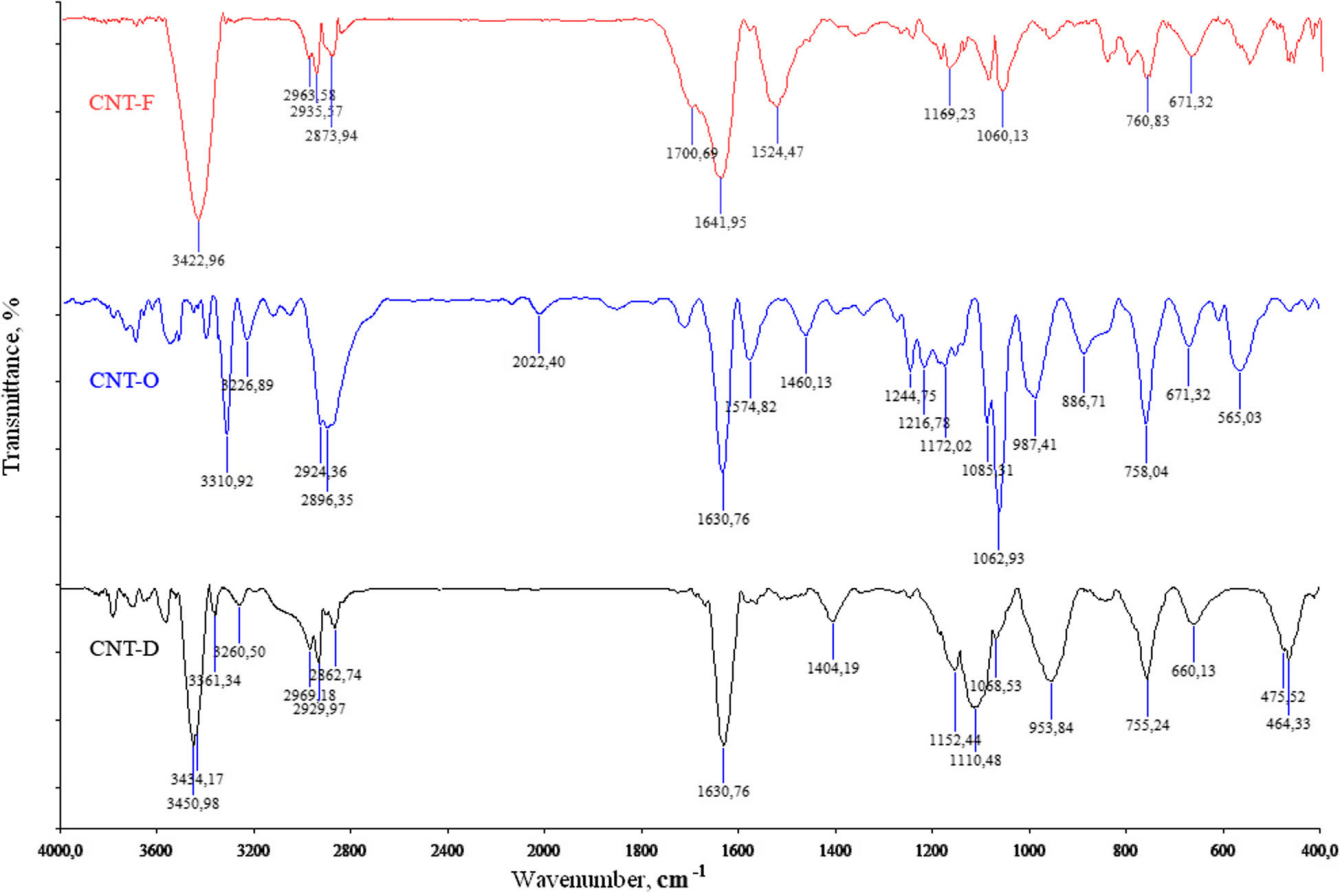
Infra-red spectra of CNTox (CNT-O), CNT-DOX (CNT-D), CNT-FITC (CNT-F)

The bindings between CNTox and doxorubicin (DOX) and fluorescein (FITC) were estimated on the basis of infrared spectral data as demonstrated in Fig. 4.

The presence of a strong band at υ = 3311 cm^−1^ (CNT-O) and υ = 3451 cm^−1^ (CNT-D) attributed to the CON-H coupling fluctuations, as well as the presence of a strong band at υ = 1634 cm^−1^ (CNT-O) and υ = 1631 cm^−1^ (CNT-D) attributed to the O-CNH bonding vibrations, which clearly indicates the type of amide bond between CNT and Dox. Also, IR showed the absorption of C-H bonds which is located at 2969–2834 cm^−1^ and a broad band at 1460–1407 cm^−1^ from the C-O-H fragment. Thus, we can conclude that as a result of chemical reactions, CNTs were functionalized with an antitumor drug (doxorubicin) and a fluorescent label (FITC).

### Cytotoxicity of CNTs at Different Stages of Functionalization on Monolayer and Spheroids Cell Growth Models

**Fig. 5:**
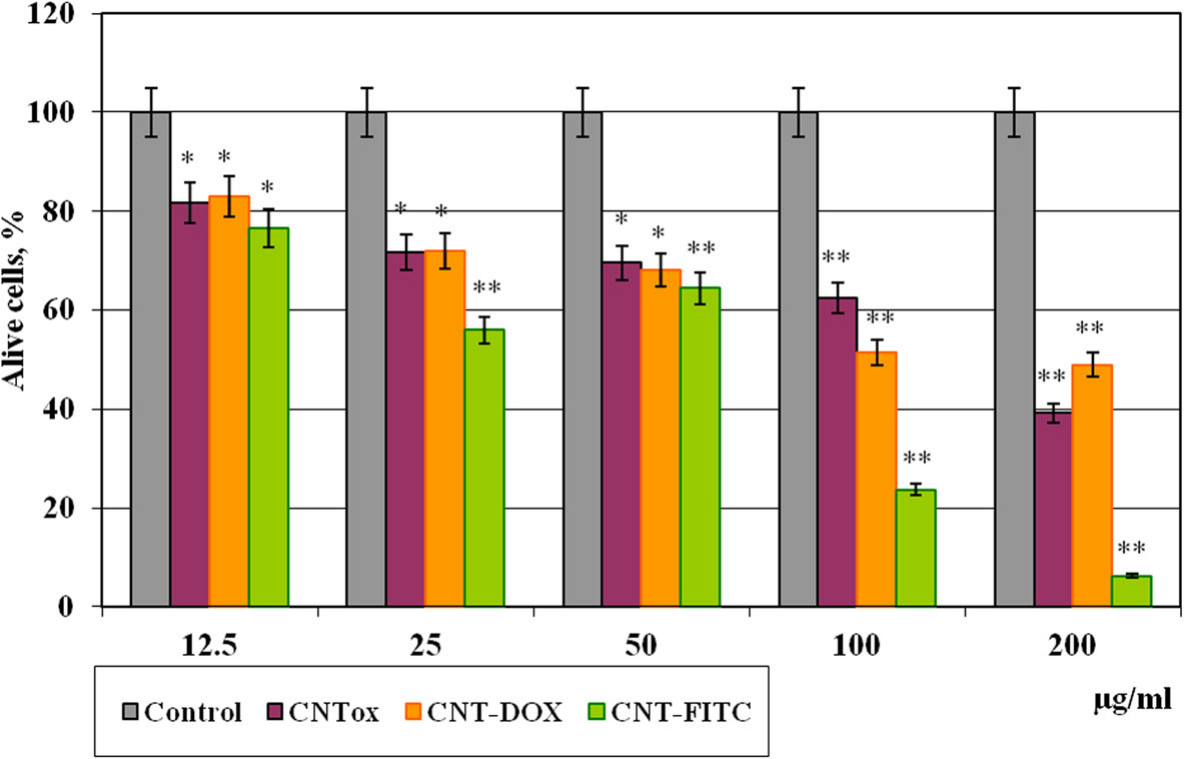
Viability of tumor cells HT29 in monolayer culture after incubation during 48 h with CNTs and its derivatives (CNT-Dox and CNT-FITC). Statistical significance: *р ≤ 0.05 or **р ≤ 0.01

The next step was to determine the cytotoxicity of oxidized CNTs (CNTox), CNTs functionalized with doxorubicin (CNT-Dox), and fluorescent label (CNT-FITC). The results of the MTT test on monolayer culture of HT29 are shown in Fig. 5.

As a result, it was demonstrated that CNTox, CNT-Dox, and CNT-FITC have a moderate cytotoxic effect at concentrations of 12.5 μg/ml (81%). Increasing CNTox concentration from 25 to 50 and 100 μg/ml led to dose-dependent decreasing the viability of tumor cells to 71.8–69.6–62.5% accordingly compared with control. At concentration of CNTox up to 200 μg/ml, the viability of HT29 decreased to 39.2%. At that time, CNT-Dox at low concentrations (12.5–50 μg/ml) did not show statistically significant cytotoxicity, compared with CNTox. At high concentrations (200 μg/ml), CTN-Dox had even less cytotoxicity than CNTox (50%). At the same time, CNTs, functionalized with fluorescein, had a relatively higher cytotoxic effect on tumor cells. After incubation with CNT-FITC in concentration of 25 μg/ml, HT29 cell survival reduced to 55% and at 100–200 μg/ml to 23 and 7% respectively. Thus, in that case, CNTs played the role of rather inert cell substrate. More than that, CNTs immobilized Dox and reduced Dox cytotoxicity. In previous studies [33], we have demonstrated that primary nanotubes have hydrophobic qualities, moderate cytotoxic effect, and stimulate the formation of a large number of cellular aggregates. According to the literature [39], surface charge of CNTs changes during the oxidation. CNTs become more hydrophilic, and it leads to the formation of smaller aggregates and increase CNTs cytotoxic effect. The data we receive in previous study confirms this tendency. CNTox reduced survival of tumor cells by at least 60% compared to control, CNT-Dox at 45%, and CNT-FITC at 97% (at 200 μg/ml). Explanation of the obtained data may be in reports of the other authors that functional ligands, in most cases, change the surface zeta potential of CNTs, stimulate solubility of CNTs, and increase cell penetration of CNTs [40]. The ability of CNTs to form aggregates after oxidation and functionalization decreases, while the permeability through the cell membrane increases and the reactivity of cellular organelles increases. More than that, CNT-Dox, at high concentrations, has lower cytotoxic effect than CNTox. At the same time, according to the obtained data, it can be assumed that the peptide bond keeps doxorubicin on the surface of the CNT. Peptide bond between CNTs and Dox is sufficiently stable in cell culture conditions. It does not break down and take doxorubicin in inactive form. At the same time, fluorescein-sodium molecular (C_20_H_12_O_5_), a ligand of the size of 332.311 g/mol, quite easily dissociates from the surface of the CNTs and enters the cells. In results, there are irreversible violations of DNA structures, nuclei, and mitochondria [41].

**Fig. 6:**
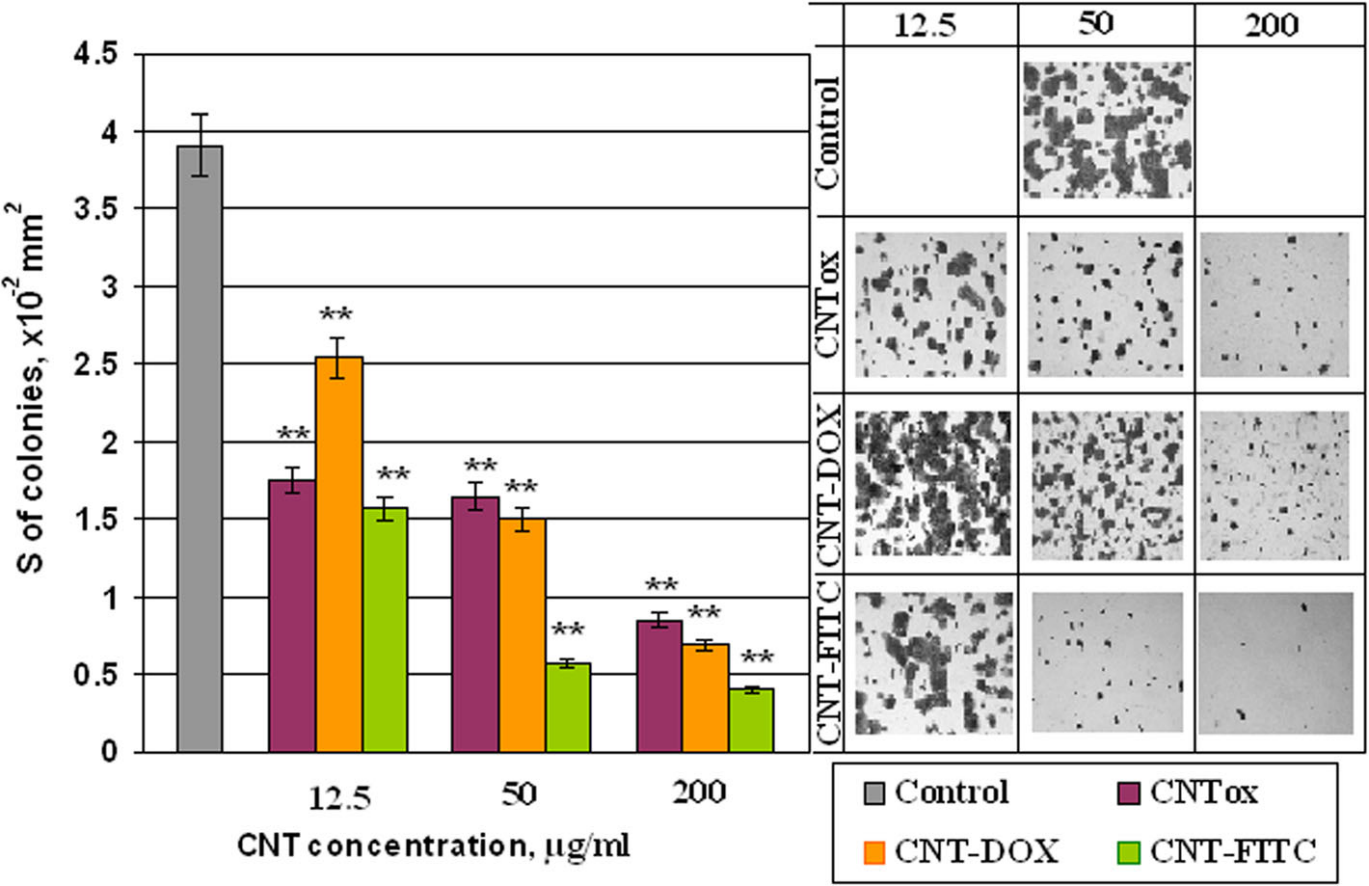
The area of colonies of HT29 tumor cells in monolayer culture which was incubated with CNTox (# 1), CNT-Dox (# 3), CNT-FITC (# 5). Statistical significance: *р ≤ 0.05 or **р ≤ 0.01

**Fig. 7:**
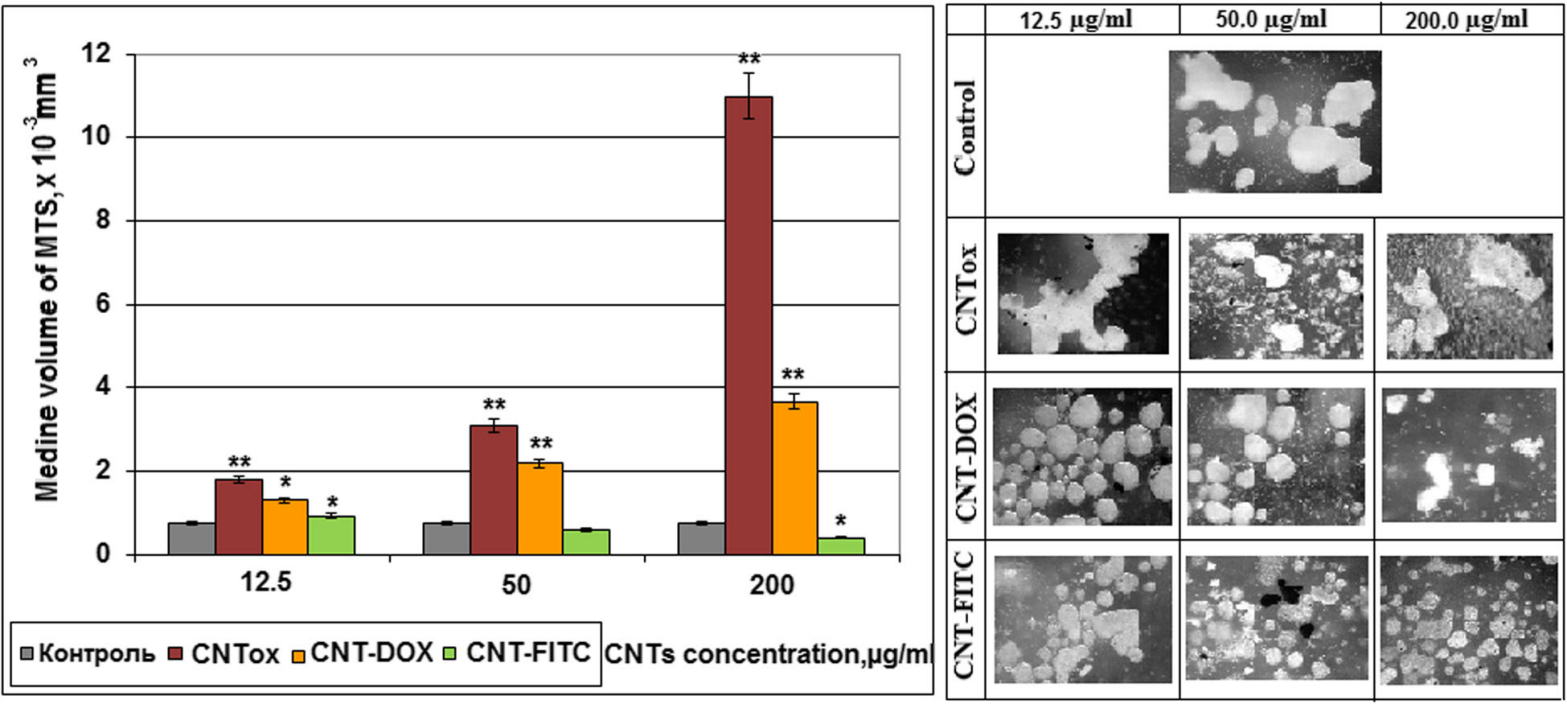
The median of multicellular tumor spheroids volume. MTS were generated in presence of CNTox (sample #1), CNT-Dox (sample #3), CNT-FITC (sample #5). Statistical significance: *р ≤ 0.05 or **р ≤ 0.01

To verify the cytotoxicity of CNTs and its derivatives and impact on adhesive properties of tumor cells, the area of the HT29 cell 2D colonies was analyzed. The results are shown in Fig. 6. It was demonstrated that CNTs and their derivatives have an influence on adhesive ability of tumor cells and formation of tumor cells colonies in monolayer culture. Incubation of tumor cells with CNTox (sample # 1) at concentrations of 12.5, 50.0, and 200 μg/ml led to dose-dependent decreasing of 2D cell colonies at 55.2%, 57.8%, and 78.3% accordingly, compared with control. At the same time, the sample of CNT-Dox (sample # 3) at the same concentrations did not cause such effect. The area of 2D colonies reduced by 34.8%, 61.6%, and 82.4% respectively. The CNT-FITC (sample #5) demonstrated the largest influence on tumor cells in monolayer culture. The area of 2D colonies after incubation with CNT-FITC decreased at 59.8%, 85.2%, and 89.8%, respectively. It should be noted that the results obtained have the same tendency as MTT-assay. But the results of the MTT analysis did not show such a significant cytotoxic effect. The resulting discrepancies may be the result of non-cytotoxic effects of CNTs on the cell colony, and, partly, anti-adhesive influence. Previously, we have demonstrated that CNTs have less cytotoxic ability then anti-adhesion. According to our data, CNTs stimulate migration of tumor cells into suspension fraction and formation of multicellular tumor spheroids (MTS). The sensitivity of cells to antitumor agents in monolayer and spheroid cultures is different. That is why on the next step, we investigated the formation of MTS by tumor cells HT29 in the presence of CNTox, CNT-Dox, and CNT-FITC. The concentrations of CNTs were the same as in previous experiments. In Fig. 7, it was demonstrated that CNTox (sample # 1) are able to stimulate the formation of MTS. Increasing of CNTox concentration is accompanied with dose-dependent increasing of median volume of MTS. At concentration CNTox from 12.5, 50, 200 μg/ml, median volume of MTS increases from 1.79 to 2.18 and 10.98 mm^3^; it is almost ten times. Derivatives of CNTs do not cause such effect on tumor cells. CNT-Dox (sample # 3) stimulates formation of MTS in 2.83 times. At the same time, incubation of 3D tumor cell culture with CNT-FITC (sample # 5) leads to a 2.4-fold decrease of MTS volume.

It is noteworthy that in most experiments, CNT-Dox has not such cytotoxic effects on tumor cells, both in 2D and in 3D culture, which could be compared with the effect of a single doxorubicin. The mechanism of cytotoxic action of doxorubicin is based on penetration into the cell nucleus and intercalation between nucleotide pairs, violation of replication and DNA repair, protein synthesis and, as a result, cell death. The cause of the reduction of cytotoxic effect of the CNT-Dox may be the binding of the doxorubicin with CNT surface through peptide bonds. It prevents Dox dissociation from CNT surface and Dox penetration into the cell and cellular organelles. This fact may let to deliver the compound to certain tissues without causing a negative effect on “non-target” objects.

**Fig. 8:**
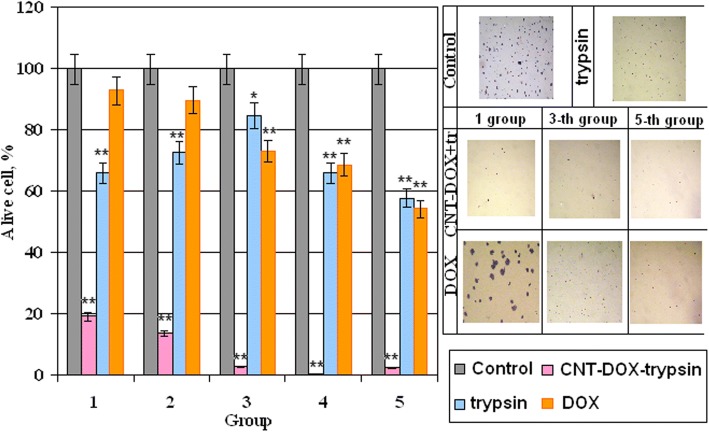
Percentage of alive cells HT29 after 48 h of incubation in the presence of trypsin, doxorubicin, CNT-Dox. Statistical significance: *р ≤ 0.05 or **р ≤ 0.01

In this case, the next step of the study was controlled release of doxorubicin. For peptide bonds breaking, we used the commonly known peptidase trypsin as the release agent. The effect of trypsin on the Dox release from the surface of the CNT was investigated by spectral analysis. Since the free doxorubicin has a fluorescence peak at 495 nm, bounded doxorubicin has no such ability. It was analyzed how the increasing of trypsin concentration affected on concentration of free doxorubicin. The results are shown in our previous work [42]. Shortly, it was demonstrated that increasing the concentration of 0.05% trypsin from 11 to 20% in culture medium contributed to an increasing the concentration of free doxorubicin from 3.13 to 6.55 μg/ml. A further increasing the concentration of trypsin to 60% did not lead to increasing in free doxorubicin. However, the concentration of trypsin by about 66% stimulated release again and led to increasing the concentration of doxorubicin in two times, to 11.38 μg/ml. Thus, it was concluded that 0.05% trypsin has several effective concentrations. Under these conditions, peptide bonds between doxorubicin and CNT were broken and doxorubicin was released. Therefore, to analyze how CNT-Dox influence the tumor cells after Dox release, we incubated tumor cells in the w/o fetal bovine serum (FBS) nutrient medium with trypsin and in the presence of CNT-Dox. The survival of tumor cells was determined using the MTT test. The ratio of the nutrient medium, trypsin, concentration of CNT-Dox, and doxorubicin are given in Table 2. The results of incubation HT29 during 48 h are demonstrated on Fig. 8. Cell viability in the presence of trypsin—blue columns, alone doxorubicin—orange columns, and CNT-Dox with trypsin—pink columns. In results, it has been found that the simultaneous use of trypsin and CNT-Dox significantly increased the cytotoxic effect of CNT and doxorubicin compared to the separately use of these substances. So doxorubicin alone at concentrations from 0.05 to 1.0 μg/ml causes a dose-dependent decreasing the percentage of living HT29 cells from 7.18 to 45.7% relative to control (Fig. 8, orange columns). Incubation of CNT-Dox at concentrations of 20.0 to 1.25 μg/ml with trypsin at concentration from 0 t0 70% led to a dose-dependent decreasing in the percentage of living cells from 80.9 and 99.8% respectively (Fig. 8, pink columns). At the same time, incubation with trypsin alone leads to decreasing percentage of living cells only by 34.0–42.0% (Fig. 8, blue columns). Thus, we can conclude that we observe the synergistic effect of CNT-DOX and trypsin. Each individual component has a small cytotoxic effect on cells, and together the cytotoxic potential of substances increases several times.

**Table 2:** Concentrations of trypsin, Doxorubicin, CNT-DOX, and nutrient medium for MTT-assay, after incubation during 48 h

Groups	DMEM w/o FBS, μl	Trypsin in DMEM w/o FBS, μl	CNT-Dox, μg/ml	Doxorubicin, μg/ml
1	100	0	20	0.05
2	50	50	10	0.125
3	45	55	5	0.25
4	38	62	2,5	0.5
5	30	70	1,25	1.0

As functional groups (Dox) were attached to CNTs surface by peptide bonds as, in vivo Dox release will be realized in organs of the gastrointestinal tract with an increased content of proteases, peptidases. In organism, proteases are used for various metabolic processes. Acid proteases secreted into the stomach (such as pepsin) and serine proteases present in duodenum (trypsin and chymotrypsin) enable us to digest the protein in food [43]. Other proteases are present in leukocytes (elastase, cathepsin G) and play several different roles in metabolic control. This is one of the fastest “switching on” and “switching off” regulatory mechanisms in the physiology of an organism. By complex cooperative action, the proteases may proceed as cascade reactions, which result in rapid and efficient amplification of an organism’s response to a physiological signal. Therefore, the authors suggest that CNT-Dox construction will be the most effective in the case of stomach, pancreas, liver, and small intestine cancer localization in case of parenteral administration of drug.

### Influence of CNTs and Their Derivatives on Cell Blood Composition, Hepatic Enzyme System, and Proteins Turnover In Vivo

**Fig. 9:**
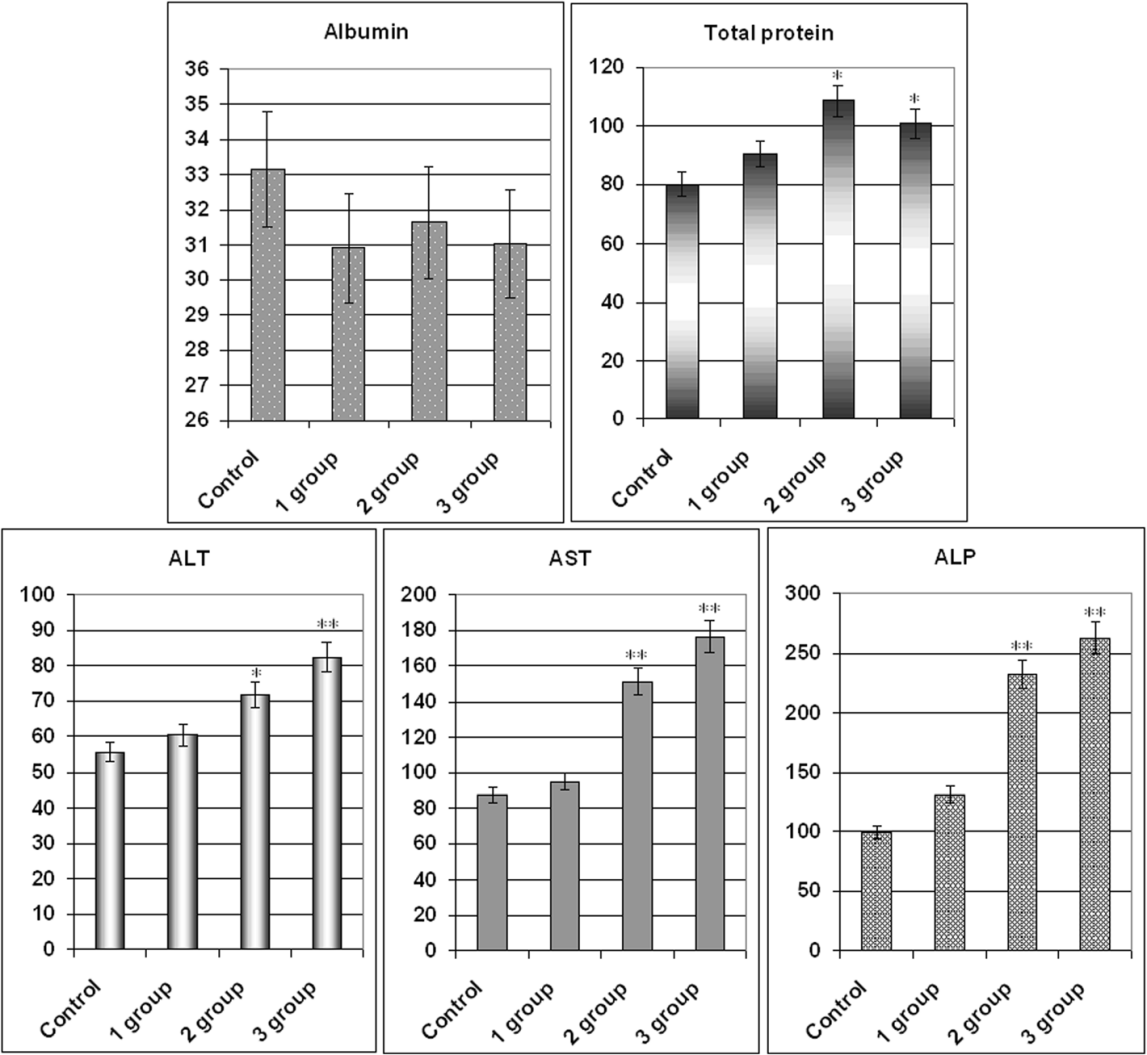
Monitoring of clinical parameters following CNTox (1 group), CNT-DOX (2 group), free DOX (3 group) administration in Balb/2a mice. Mice were treated by CNTox, CNT-DOX, DOX every 3 days during 4 weeks, in concentrations: CNT—1.5 g/kg and Dox—20 mg/kg of weight. Each experiment was done in triplicate. Statistical significance: *р ≤ 0.05 or **р ≤ 0.01. *ALT* alanine transferase, *AST* aspartate transferase, *AP* alkaline phosphatase

To determine the impact of the CNTox and CNT-Dox on the protein metabolism and the state of the hepatic enzyme system, the level of albumin (Al), total protein (Tp), alanine aminotransferase (ALT), aspartate aminotransferase (AST), and alkaline phosphatase (ALP) was determined. Data are given in Fig. 9a, b. As a result, it was noted that CNT-Dox and Dox have influence on the hepatic enzymes activity, namely on the AST and ALP. AST level increased in mice serum from 1 group for 21.6%, 2 group for 93.9%, and 3 group for 126.4% compared with control. At the same time, the activity of ALP increased in mice serum from 1 group for 23.5%, 2 group for 119.1%, and 3 group for 147.8%. CNTox administration did not show statistically significant changes in AST, ALT, and ALP levels. In addition, it should be noted that CNTox, Doxorubicin, and CNT-Dox slightly increased the level of total protein in the blood of experimental animals. So, we found that systematic introduction of CNTox have not influence on mice serum enzyme profile. And opposite, administration of CNT-Dox and Dox had toxicity influence and induced a chronic hepatic damage. Notably, symptoms of hepatitis had more manifestation after Dox treatment than CNT-Dox. To determine possible inflammatory processes and systemic effects of CNTox, CNT-Dox on the state of blood cells composition, it was analyzed the blood cell formula of the experimental animals. The results are shown in Table 3. Obtained data are in good agreement with the well-known manifestations of doxorubicin’s hematological toxicity: anemia (decreased red blood cells, hemoglobin, hematocrit), thrombopenia (platelet count), neutropenia (decrease in the number of granulocytes). Interestingly, that it was demonstrated the same direction of the changes of practically all hematological parameters in the 1 (CNTox), 2 (CNT-Dox), and 3 (Dox) groups of animals. However, the change in the 2 and 3 groups of animals was statistically significant accordingly to control and 1 group. In the 2 group (CNT-Dox), it was found pronounced decreasing of the monocyte content related to the system of phagocyte mononuclear cells and cell’s immune response (9.5% and 0.12 × 10^9^/l in control, 4.6% and 0.06 × 10^9^/l in 2 group, and 4.55 and 0.05 × 10^9^/l in 3 group). Animals of 2 and 3 groups demonstrated reducing the content of granulocytes (neutrophils) (12.6 and 0.16 × 10^9^/l in control and 8.5% and 0.2 × 10^9^/l in 2 group and 8.05 and 0.16 × 10^9^/l in 3 group). The number of lymphocytes, both in percentage and absolute, increased in 2 and 3 groups (77.9% and 0.97 × 10^9^/l in control and 86.9% and 2.0 × 10^9^/l in 2 group and 87.8% and 2.3 × 10^9^/l in 3 group). The main function of the lymphocytes is recognition of the antigen and participation in the adequate immunological response of the body. T lymphocytes perform regulatory and effectors functions. B lymphocytes take part in humoral immunity, providing immunoglobulins in response to stimulation of other people’s antigens. So, it can be assumed that an increase in the index of lymphocyte content in experimental 1, 2, and 3 groups can be associated with the introduction of foreign antibodies (CNTs) and/or tissue distraction [44]. Some decrease in the absolute number of leukocytes in 2 (1.2 × 10^9^/l) and 3 (0.85 × 10^9^/l) groups compared with the 1 group of animals (2.0 × 10^9^/l) can be regarded as the result of a gradual accumulation of the reversal of toxic effects of Dox. It is described the reaction of WBC in experimental animal groups as well-known toxic hematologic impact from doxorubicin in the development of leucopenia. The analysis of indicators reflecting the number and state of RBC (erythrocytes and hemoglobin) also showed the same trend of misbalance in the 2 and 3 groups of animals. Statistically significant thrombocytopenia and anemia are more indicative in animals of 2 and 3 groups: the number of erythrocytes (5.54 × 10^12^/l in control, 3.97 × 10^12^/l in 2 group, and 3.12 × 10^12^/l in 3 group), the amount of hemoglobin (78 g/dl for intact animals, 55.5 g/dl for 2 group, and 46.2 g/dl for 3 group), hemoglobin (25.25% for intact animals and 16.75% for 2 group and 15.12% for 3 group). The amount of erythrocytes decreased but the average content of hemoglobin in one erythrocyte did not decrease. And, as a result, concentration of hemoglobin per one erythrocyte increased. Analysis of the RBW counts let us suggest that in 2 and 3 group of animals, there are several processes: decreasing the formation of erythrocytes in the bone marrow, the acceleration of erythrocyte destruction, the violation of the structure of membranes of red blood cells, and molecular defects (oxidation) of hemoglobin. Thrombocytopenia (27 × 10^9^/l in intact animals, 14.0 × 10^9^/l in 2 group, and 12.05 × 10^9^/l in 3 group), a dose-dependent reversible myelosuppression, leucopenia and granulocytopenia (neutropenia) are the predominant manifestations of doxorubicin hematologic toxicity and is the most common acute dose-limiting toxicity of this drug. The direction of changes in the platelet content in the blood of experimental animals treated with CNTox and CNT-Dox is the same; however, significantly more pronounced in the group of animals receiving free Dox.

**Table 3:** Composition of the formed blood elements in the experimental animals after 4 weeks of administration of CNTox and CNT-Dox

	Control	CNTох	CNT-Dox	Dox
Leukocytes, 10^9^/l	1.25	2.0	1.2	0.85
Lymphocytes, 10^9^/l	0.97	1.75	2.0	2.3
Lymphocytes, %	77.9	86.7	86.9	87.8
Monocytes, 10^9^/l	0.12	0.09	0.06	0.05
Monocytes, %	9.5	7.15	4.6	4.5
Granulocytes, 10^9^/l	0.15	0.20	0.20	0.16
Granulocytes, %	12.6	9.15	8.5	8.0
Hemoglobin, g/dl	78.0	72.5	55.5	46.2
Erythrocytes, 10^12^/l	5.54	4.96	3.97	3.12
Hematocrit, %	25.25	21.25	16.75	15.12
Av. vol. erythrocyte	45.5	42.9	42.65	42.15
Av. hemoglobin per erythrocyte, lg	14.05	14.55	14.2	13.01
Av. concentration of hemoglobin per erythrocytes, g/dl	309.5	340.5	335.5	336.2
Thrombocytes, 109/l	27.0	25.5	14.0	12.05

Thus, according to our results from in vivo experiments, it was demonstrated that systematic introduction of CNTox have not influence on mice serum enzyme profile and protein turnover. And opposite, administration of CNT-Dox and Dox had toxicity influence and induced a chronic hepatic damage. More than that, dose-dependent reversible myelosuppression, leucopenia, and granulocytopenia (neutropenia) was shown for the group of animals receiving free Dox. This predominant manifestation of hematologic toxicity was less pronounced in the group of animals receiving free CNT-Dox and CNTs. So, we can assume that, in the case of parenteral administration of CNT-Dox under action of gastric juice peptidases, CNT-Dox particles breaks up into free CNT and Doxorubicin. After that, Doxorubicin enters to the stomach cells and partly in the bloodstream. That also causes the specified effect on blood cells composition. For modeling Dox release in vivo, trypsin were used in vitro.

One of the goals of this investigation was to minimize the side effects of carbon vehicle on the body. Previously, the authors demonstrated that CNTs may be a potential threat to tumor development due to its ability to stimulate cell migration and support cells in suspension fraction [33]. In this case, CNTs themselves play a role of artificial extra cellular matrix. Another way CNTs can stimulate suspension cells to aggregation. If this process will be coincident with antitumor drug accumulation on CNTs surface, CNTs will attract substrate-independent cells and kill them. Simultaneously, it was found that pure CNTs did not have statistically significant cytotoxicity to tumor cells. So, CNTs alone are not dangerous as cytotoxic agents and can act as carrier for antitumor drugs to target cells. Then, studies were conducted to the functionalization of the CNTs by antitumor antibodies. It was demonstrated that CNTs are able to carry on the surface not only the Dox but also specific tumor antibodies—anti EGFr [42]. Under the action of trypsin, Dox was released and realized strong cytotoxic effect on tumor cells. Recent studies have shown the synergy cytotoxic effect of CNTs and Doxorubicin after release from CNT-Dox construct in the presence of trypsin. Such effect was not demonstrated by CNTs and Dox separately. Therefore, the benefits from the use of CNTs seems in the usage CNTs as vehicle for antitumor antibody and drug. On the other hand, the negative effects of Doxorubicin, like many other early antitumor drugs, are totally cytotoxic effects. Binding of the Dox to the CNTs partially blocks it. This effect allows ensuring local accumulation of the Dox in the focus of tumor activity with subsequent release and action. Thus, greater efficacy can be achieved with a smaller dose of administration. This effect can be achieved only if there are tumor-specific antibodies on the surface of the CNTs. The possibility of creating such construct was demonstrated by the authors in previous work. Undoubtedly, that CNTs dissemination, accumulation, and Dox effective should be tested on mixed culture in vitro and on a tumor model in vivo. For this purpose, synthesized CNT-FITC was created and conducted. The ability of the CNTs to act as an extra-cellular matrix and the significance of this process for the “tumor-body” system should be tested on the animal model. The effectiveness of the drug will be investigated on the model of Ehrlich carcinoma and colon rectal carcinoma [44]. The distribution of CNTs in the tissues of the body will be investigated using the construct created by the CST-FITC, as was mentioned in this work. The accumulation of CNTs in the tissues of the organs of the gastrointestinal system, namely the stomach, liver, and intestines, will be analyzed by histological assay. These studies are already conducted by the authors.

According to Shang-Lin Wang [4] and our data, free doxorubicin causes similar side effects, but more pronounced than CNT-Dox. Other authors reported that toxicity effect of CNTs depends on way of administration, dose, and time of exposure and varies according to the size and type of the cells [45–49]. Settling of CNTs in the tissues of the liver, kidneys, and stomach with prolonged exposure can cause oxidative stress, DNA damages, compromise cell proliferation, necrosis of tissues, and chronic inflammation [50]. According to Manna [51], CNTs can cause oxidative stress and compromise cell proliferation. In the case of in vitro studies, the cytotoxicity of CNT highly depends on the degree of CNTs purification, functionalization, size, and surface charge [52–55]. According to our results and previous data, the obtained CNTs did not demonstrate a significant cytotoxic effect [33]. More than that, our data let us suggest that there is a combined cytotoxic effect of CNTs and DOX that occurs as in vitro. That is why we assumed that obtained CNTs can be used against tumor cells, in case of the target accumulation of CNTs/CNT-Dox in tumor tissue. However, mechanisms of the cytotoxic effect of CNTs are not completely clear. Some authors suggested that CNT particles activate NF-kappaB pathway depending on the dose and that the mechanism of activation was due to activation of stress-related kinases [51]. Other authors reported that CNTs have a direct pro-apoptotic effect in vitro in different cancer cell lines and tumor cells obtained from surgical specimens [56], CNTs able to modify fatty acids in cell membranes [57] or erythrocyte membrane damage [58]. For further investigation of accumulation CNTs in cells and tissue, we plan to use CNT-FITC composite.

Other unclear questions are as follows: what is the prolonged impact of CNTs/CNT-Dox on organism level and how does it stimulate accumulation CNTs/CNT-Dox in tumor tissue? To investigate the influence of chronical introduction of CNTs/CNT-Dox, long-time studies on model of transplanted or initiated tumors of various localizations will be realized. Other authors analyzed the distribution of nanotubes throughout the body of mice after pulmonary exposure [59]. In results, accumulation of MWCNTs was documented in several organs, including notably the white pulp of the spleen and the bone marrow. The CNTs usually deposit in the liver, spleen, or lungs after they have served their purpose from where they are expelled gradually out of the body through the renal excretion route [60]. Zhao and Liu reported that accumulation of CNTs in the body can lead to granulomatous inflammation or alveolar septal thickening. Zhuang Liu et al. reported that SWNT-PTX affords higher efficacy in suppressing tumor growth than clinical Taxol® in a murine 4T1 breast-cancer model, owing to prolonged blood circulation and tenfold higher tumor paclitaxel (PTX) uptake by SWNT delivery likely through enhanced permeability and retention [61]. Accumulation of CNTs in the target tissue can be enhanced by placing on the CNTs surface specific antibodies which over expressed by tumor cells. The possibility of using antibodies for the targeted delivery of nanostructured preparations has been described by many authors [62, 63].

## Conclusion

In 2D culture CNTox concentration from 25 to 50 and 100 μg/ml led to dose-dependent decreasing the viability of tumor cells to 71.8–69.6–62.5% accordingly compared with control. At concentration of CNTox up to 200 μg/ml, the viability of HT29 decreased to 39.2%. At that time, CNT-Dox at concentrations 12.5–25–50 μg/ml did not show statistically significant cytotoxicity, compared with CNTox. At high concentrations (200 μg/ml), CTN-Dox had even less cytotoxicity than CNTox (50%). After incubation with CNT-FITC in concentration of 25 μg/ml, HT29 cell survival reduced to 55% and at 100–200 μg/ml to 23 and 7% respectively. In 3D culture, increasing of CNTox concentration is accompanied with dose-dependent increasing of median volume of MTS. At concentration CNTox from 12.5, 50, 200 μg/ml median volume of MTS increases from 1.79 to 2.18 and 10.98 mm^3^. CNTs-Dox and CNT-FITC did not cause such effect on tumor cells. Doxorubicin alone at concentrations from 0.05 to 1.0 μg/ml causes a dose-dependent decrease in the percentage of living HT29 cells from 7.18 to 45.7% relative to control. Incubation CNT-Dox at concentrations of 20.0 to 1.25 μg/ml with trypsin at concentration from 0 to 70% led to a dose-dependent decreasing the percentage of living cells from 80.9 and 99.8% respectively. At the same time, incubation with trypsin alone leads to decreasing percentage of living cells only by 34.0–42.0%. So, the synergistic effect of CNT-DOX and trypsin was observed. Each individual component has a small cytotoxic effect on cells, and together the cytotoxic potential of substances increases several times. In vivo, systematic introduction of CNTox have not influence on mice serum enzyme profile. And opposite, administration of CNT-Dox and Dox had toxicity influence and induced a chronic hepatic damage, thrombocytopenia, a dose-dependent reversible myelosuppression, leucopenia, and granulocytopenia (neutropenia). Notably, symptoms of hepatitis had more manifestation after Dox treatment than CNT-Dox.

So, the possibility of targeted delivery and controlled release of any highly toxic drug with the use of CNT depends on strategies of reducing toxicity of CNTs and realizing potential of CNTs and anti-tumor drugs “in right place in right time.” According to the data obtained by authors and literature, it is possible. Our data support the assumption that this approach allows to reduce toxicity of the doxorubicin on the general biochemical indicators of blood and violations in the blood cells composition. At the same time, doxorubicin releasing is realized under certain conditions. And combined effect of CNTs and doxorubicin let us achieve greater efficacy in suppressing tumor cell growth in vitro.

## Abbreviations

Al: Albumin
ALP: Alkaline phosphatase
ALT: Alanine aminotransferase
AST: Aspartate aminotransferase
CNT-Dox: Carbon nanotubes which were functionalized by doxorubicin
CNT-FITC: Carbon nanotubes which were functionalized by fluorescein
CNTox: Oxidized carbon nanotubes
CNTs: Carbon nanotubes
Dox: Doxorubicin
FTIR: Fourier-transform infrared spectroscopy
MTS: Multi-cellular tumor spheroid
MWCNTs: Multi-walled carbon nanotubes
PTX: Paclitaxel
Tp: Total protein
